# Impact of dielectric barrier discharge cold plasma on the lipid oxidation, color stability, and protein structures of myoglobin-added washed pork muscle

**DOI:** 10.3389/fnut.2023.1137457

**Published:** 2023-02-09

**Authors:** Xiaoting Wang, Jin Wang, Zhaobin Wang, Wenjing Yan, Hong Zhuang, Jianhao Zhang

**Affiliations:** ^1^College of Food Science and Technology, National Center of Meat Quality and Safety Control, Collaborative Innovation Center of Meat Production and Processing, Quality and Safety Control, Nanjing Agricultural University, Nanjing, China; ^2^College of Food and Drug, Luoyang Normal University, Luoyang, China; ^3^Key Laboratory of Environmental Medicine and Engineering, Ministry of Education, Department of Nutrition and Food Hygiene, School of Public Health, Southeast University, Nanjing, China; ^4^Quality and Safety Assessment Research Unit, U.S. National Poultry Research Center, USDA-ARS, Athens, GA, United States

**Keywords:** dielectric barrier discharge cold plasma, bioactive compounds, myoglobin, lipid oxidation, secondary structure, novel non-thermal processing technology

## Abstract

Cold plasma has been considered a novel non-thermal processing technique and attracted a high attention by the food industry. In this study, the influences of dielectric barrier discharge cold plasma (DBD-CP) on the myoglobin (Mb)-added washed pork muscle (WPM) were evaluated. The electrophoresis pattern, autoxidation, and secondary structure of Mb were analyzed. The results found that DBD-CP caused the decrease of the redness and total sulfhydryl (T-SH) in WPM, while the increase of non-heme, peroxide value (PV), and thiobarbituric acid reactive substances (TBARS), suggested that treatment triggered protein oxidation and heme degradation. Additionally, DBD-CP treatment enhanced the autoxidation of Mb, induced the release of intact heme from the globin, rearranged the charged groups, and promoted Mb aggregation. The transformation of α-helix into the random coil of Mb demonstrated that DBD-CP weakened the tensile strength. Overall, data indicated that DBD-CP promoted autoxidation and changed the secondary structure of Mb, accelerating Mb-mediated lipid oxidation in WPM. Thus, further studies about the optimization of processing conditions by DBD-CP need to be performed.

## 1. Introduction

During meat processing and storage, lipid oxidation is the main reason causing the deterioration of meat or relevant products, which easily leads to adverse effects on the color attributes, flavor, taste, nutrients, and safety (1). Therefore, lipid oxidation of meat is getting more and more attention by the meat industries and scientists. Furthermore, it is becoming more important to restrain the lipid oxidation in meat due to the increasing demand for pre-cooked meat (2).

Dielectric barrier discharge cold plasma (DBD-CP) is a novel processing technology used in agricultural products, which has drawn widespread attention and concern in the food industry because of its energy-saving and environmental characteristics (3). Cold plasma (CP) is composed of ions, atoms, electrons, and reactive oxygen/nitrogen species (ROS/RNS) (4). These active substances in plasma can quickly and effectively inactivate microorganisms in the native environment or cause changes in the food functional substances of food. Wang et al. (5) reported that free radicals produced during CP processing promote lipid oxidation and affect meat color and other quality characteristics. The mechanisms of lipid oxidation induced by CP are not fully understood.

Myoglobin (Mb) and hemoglobin (Hb) are the main heme proteins in muscle food, which contribute to the redness of meat. They are also essential endogenous factors that induce lipid oxidation in the muscle. For red muscles, Mb is the main heme protein accounting for more than 50–70% of total heme protein in pork muscle and 65–90% of total heme protein in beef muscle (6). Recently, research on the effect of the redox of Mb and metalloproteins has been paid more and more attention, such as high hydrostatic pressure (7), X-ray (8), and low-frequency magnetic field (9), but with little about CP field. However, most studies focus on the relationship between CP and proteins' structural and functional properties. The mean diameter of zein micelles decreased, and the solubility in non-alkaline solution increased after CP treatments (10). Nyaisaba et al. (11) explored the squid proteases and gel properties using atmospheric CP to assess their effects on the proteins' enzyme activity and chemical properties. Luo et al. (12) illustrated that the ROS and RNS produced by DBD-CP have significant influences on the structural and chemical properties of myofibrillar proteins (MPs) from dry-cured bacon, which decreased the α-helix and increased random coils of proteins. Furthermore, cold plasma improved the emulsion capacity and foaming capacity of MPs extracted from *Longissimus Dorsi* muscle after treatment for 10 min (13). A coordinated water molecule in deoxy (Fe^2+^) Mb or ligands such as O_2_ and NO bind the iron atom of the heme ring moiety of reduced Mb affects the onset of Mb oxidation (14). Because of its particular structure, the redox state of Mb is unstable and susceptible to the influence of ROS produced by cold plasma to induce lipid oxidation. However, the mechanism has not been systematically studied up to now.

Herein, this study investigated the addition of Mb to washed pork muscle under different DBD-CP treatments (0, 50, 60, 70 kV). The various indexes of meat samples, including surface color, non-heme iron content, total sulfhydryl, and lipid oxidation were determined during storage. Meanwhile, changes in the structure of Mb treated with DBD-CP were explored by analyzing the Mb oxidation, polyacrylamide gel electrophoresis, absorption spectra, particle size, zeta potential, and circular dichroism spectra.

## 2. Materials and methods

### 2.1. Samples preparation

The myoglobin (95–100%, Sigma Aldrich USA) used in this study was extracted from equine skeletal muscle. Since Mb was unstable and prone to be oxidized, highly oxidized Mb (>95% of metmyoglobin) would affect the experimental process. Therefore, Mb was reduced by sodium hydrosulfite solution under the atmosphere filled with nitrogen (15). The reduced Mb was dissolved in deionized water to prepare a specific solution concentration in the nitrogen-filled surrounding and stored under 4°C for standby application.

### 2.2. Preparation of washed pork muscle

Fresh pork longissimus lumborum (LL) was purchased from a local market, immediately shipped to the laboratory under cold storage. Washed pork muscle (WPM) was prepared as described formerly (16). Before the treatments, fresh pork LL was kept at −40°C for 1 d to remove heme proteins better. The pork LL was minced. Then the minced muscle was mixed with sodium phosphate buffer (m/v,1:4), homogenized, and centrifuged to collect precipitation. This process was repeated five times, and the collected precipitate was called WPM. The whole washing process was carried out at a low temperature (4°C). After pre-washing, about 0.2 g WPM was used to determine the moisture content of samples. The remaining WPM was vacuum-packaged in polyvinylidene chloride material and stored at −40°C for further tests.

### 2.3. Addition of Mb to WPM

According to the previous method, myoglobin was added to washed pork muscle, then stirred well with a glass rod with minor changes (17). Briefly, except for the control groups, the final concentration of Mb was 40 μmol/kg WPM. If necessary, deionized water was used in the system to regulate the moisture content to 88%. The pH of WPM was adjusted to 7.0 with 1 N HCl, then the addition of streptomycin sulfate (200 ppm) to the system restrained the growth of microorganisms. Sodium phosphate buffer (50 mM, pH7.0) was added to WPM instead of myoglobin solution as the control. The mixture samples were stored on an ice bed for DBD-CP treatment. The WPM containing Mb was called WPM-Mb.

### 2.4. Properties of WPM

The DBD device used in this paper was the same as Huang et al. (18) described. About 10 g sample was placed in Petri dishes of 50 mm diameter and then subjected to DBD-CP treatments. The plasma source comprised two aluminum disc electrodes with a diameter of 150 mm, over two 2 mm thick layers of polypropylene between which the samples were placed. The distance between the two electrode plates was 35 mm. DBD-CP treatment was carried out at 0, 50, 60, and 70 kV in air atmospheric for 180 s at room temperature. The operating frequency of the system was 50 Hz. After processing, the petri dishes were rapidly placed at 4°C and measured during the pre-determined time (day 0, 1, 2, 3, and 4). The untreated WPM with Mb was used as the control. All treatments had 3 replicates per storage time.

#### 2.4.1. Color attributes

CIE-Lab redness (a^*^) values of washed pork muscle were determined by Chroma meter (Model CR-400; 8 mm diameter aperture, Illuminant D65, 2° observer; Konica Minolta, Japan). This instrument was adjusted with a white correcting board before measuring. Six different points were selected from the sample for determination, and the results were averaged.

#### 2.4.2. Non-heme iron content

Non-heme iron content was evaluated by the previously described method with minor modification (19). A 1.0 g sample was added to a centrifuge tube followed by a 3 mL buffer solution (0.1 M sodium citrate, 0.1 M phosphate, pH 5.5). The mixture was centrifuged at 1800 g for 10 min. Next, 1 mL supernatant was mixed with 0.5 mL ascorbic acid (2% in 0.2 N HCl, w/v), and trichloroacetic acid (TCA) (11.3%, w/v), respectively. The mixture was centrifuged again at 3000 g for 15 min. Subsequently, 1 mL supernatant, 10% ammonium acetate (0.4 mL), and 0.1% ferrozine color reagent (0.1 mL) were mixed, then placed at room temperature for 15 min. The non-heme iron content was calculated from the ferric chloride standard curve determined by the absorbance at 562 nm.

#### 2.4.3. Peroxide value

Sample (0.5 g) was added to a chloroform/methanol mixture (1:1, v/v, 5 mL), then homogenized for 40 s using a Polytron Type PT 10/35 (Brinkmann Instruments) at 9000 rpm and poured into a tube (20). This Polytron was washed with another 5 mL of the mixture solvent for 20 s and then poured into the corresponding tubes. Afterward, 3.08 mL NaCl (0.5%, w/v) was added to the tubes and centrifugated at 1800 g for 5 min. Two phases were separated in the tube. The 2 mL lower layer liquid was moved into a new centrifugation tube. Then the mixture solvent (1.33 mL), ammonium thiocyanate (30%, w/v, 25 μL), and ferrous chloride (25 μL, 18 mM) were added to corresponding tubes, respectively. These tubes were placed at room temperature to avoid light for 20 min. The standard curve of cumene hydroperoxide was established based on the absorbance of the samples at 500 nm to calculate PV.

#### 2.4.4. Thiobarbituric acid reactive substances

TBARS is one of the crucial indicators to judge the secondary oxidation of lipid. According to the method described by Richards et al. (20), sample (0.2 g) was added into a tube, and then a 1.2 mL mixture of 50% TCA with 1.3% 2-Thiobarbituric acid (TBA) was added (20). After being shaken for 15 s by a vortex oscillator, the tube was incubated at 65°C for 1 h. Next, the tube was cooled at 4°C without light for 1 h. After centrifugation at 16,100 g and 4°C for 6 min, the absorbance was determined at 532 and 600 nm. Finally, A_532nm_-A_600nm_ was taken as the actual absorbance of the samples. The standard curve of 1,1,3,3-tetramethoxypropane was used to calculate TBARS.

#### 2.4.5. Total free sulfhydryl (T-SH) content

Total sulfhydryl content was evaluated by Total Sulfhydryl Group Content Assay Kits (Solarbio, Beijing, China) (5,5'-dithiobis-2-nitrobenoic acid, DTNB method). Specifically, WPM (0.1 g) was added into the extracting solution (1 mL) and centrifuged at 8,000 g for 10 min. The supernatant (40 μL) and reagent one (150 μL) were added to the test and control tube, respectively. Then reagent two (10 μL) was added to the test well, 10 μL deionized water was added to the control well and incubated at 25°C for 10 min. A standard curve of glutathione was established according to the absorbance of 412 nm to calculate the concentration. The total sulfhydryl content was expressed as μmol^·^g^−1^ of muscle.

### 2.5. Properties of myoglobin

A 10 mL Mb solution prepared as described in Section 2.1 was placed in a petri dish with a diameter of 50 mm and then subjected to DBD-CP treatment. DBD-CP treatment was performed at 0, 50, 60, and 70 kV in air atmospheric for 180 s at room temperature. The operation frequency of the system was 50 Hz. After processing, the Mb solution were rapidly placed at 4 °C for further experiments.

#### 2.5.1. Sodium dodecyl sulfate-polyacrylamide gel electrophoresis (SDS-PAGE) analysis

According to Luo et al. (12), the SDS-PAGE analysis for Mb was measured with minor modifications. A 1 vol Mb solution was added with 3 vol SDS-PAGE sample buffer and boiled for 3 min before use. SDS-PAGE was prepared by separating gel (12%) and stacking gel (5%). Each well was loaded with 10 μL, and a protein standard was used as a molecular marker (Sangon Biotech, Shanghai, China). Subsequently, the gel was stained using Coomassie blue for 30 min and then decolorized with 40% ethanol containing 10% acetic acid for 30 min four times.

#### 2.5.2. Soret band of myoglobin

Soret band analysis of Mb solution treated with or without DBD-CP was performed. Briefly, the absorption spectra at 350–700 nm were determined by UV-2600 spectrophotometer (Shimadzu Instrument Inc., Japan). All tests were repeated three times at room temperature.

#### 2.5.3. Mb autoxidation rate

Mb automatic oxidation rate (*k*_*ox*_) is the rate of metMb generated. The UV-2600 spectrophotometer (Shimadzu Instrument Inc., Japan) was used to record the absorption spectra of Mb in the range of 500–700 nm. The determination method is slightly modified according to the previous study (21). Specifically, the Mb storage solution was diluted to 40 μM by 0.2 M sodium phosphate buffer (pH 7.0) and kept away from light at 4°C. The ratio of metMb was calculated based on the modified equation (22) as follows: [metMb] = −0.159 *R*_1_ – 0.085 *R*_2_ + 1.262 *R*_3_ – 0.520.


$$\begin{array}{l} where\;R_{1}=A_{582}/A_{525},R_{2}=A_{557}/A_{525},andR_{3}=A_{503}/A_{525}. \end{array}$$

The automatic oxidation rate (*k*_*ox*_) of Mb is expressed by the slope of metMb% production to time.

#### 2.5.4. Particle size and zeta potential

Particle size and zeta potential of Mb treated and untreated by DBD-CP were measured by the Zetasizer Naso ZS Particle and Zeta Potential Analyzer (Malvern Instrument, Ltd., England). All tests were fulfilled in triplicate at 25°C.

#### 2.5.5. Circular dichroism spectroscopy

CD spectroscopy was evaluated using the method described by Xia et al. (9). Mb solution (5.5 μM) was placed in 1 mm quartz cuvettes. The solution was scanned by the CD spectrometer (J-1500, JASCO, Japan) in the range of 260-185 nm.

### 2.6. Statistical analysis

The same batch of pork LL was used to prepare all the WPM in the study, and all determinations were carried out in triplicate with three repetitions. Statistical analysis was carried out using the Statistical Package (SPSS 26.0, SPSS Inc., USA). One-way analysis of variance (ANOVA) was utilized to evaluate the statistical difference. All data were expressed as mean ± standard deviation. Comparison of means was carried out by Duncan's multiple range test significant difference (*p* < 0.05).

## 3. Results and discussion

### 3.1. Lipid oxidation of WPM

#### 3.1.1. The redness of WPM

The changes in the a^*^-values of WPM (Control) and WPM-Mb treated with DBD-CP (0, 50, 60, and 70 kV) are shown in Table 1. The control showed a low a^*^ value (negative) and remained stable level throughout the storage (*p* ≥ 0.05). The remaining groups (0–70 kV) exhibited the highest a^*^ value at day 0. The a^*^-values at each storage point showed a decreasing trend with the processing voltage increased (0–70 kV). The decrease in redness from 5.32 (0-time, 0 kV) to 3.63 (0-time, 70 kV) demonstrated substantial changes in Mb during DBD-discharge at 70 kV, especially. For WPM-Mb, the decline in redness was related to the oxidation of Mb (deoxyMb, oxyMb) to metMb, leading to the brownish-gray color (14). Moreover, at each time point during storage, a^*^-values of WPM-Mb in the treated samples (50–70 kV) were notably lower than untreated (0 kV), illustrating that DBD-CP processing caused a rapid accumulation of metMb. The above experimental results indicated that (i) metMb, (ii) non-heme iron, (iii) intact heme released from globin, and (iv) ferryl-Mb/crosslink Mb were possible oxidative species that contributed to the DBD-CP-induced lipid oxidation. The results in Figure 3A also suggested a substantial amount of intact heme was released, forming free heme and globin (e.g., apoMb) after the DBD-CP treatment for 180 s.

**Table 1:** Changes in instrumental redness of washed pork muscle with added Mb treated by DBD-CP at different voltages during storage (4°C for 4 days).

**Variable**	**Treatments**	**Storage time (day)**				
		**0**	**1**	**2**	**3**	**4**
	Control	−1.18 ± 0.07^dA^	−1.24 ± 0.08^dA^	−1.24 ± 0.11^dA^	−1.19 ± 0.11^dA^	−1.22 ± 0.07^dA^
	0 kV	5.32 ± 0.22^aA^	5.13 ± 0.31^aAB^	5.02 ± 0.43^aAB^	4.87 ± 0.46^aAB^	4.54 ± 0.17^aB^
Redness (a^*^)	50 kV	4.60 ± 0.50^bA^	4.45 ± 0.34^bA^	4.35 ± 0.24^bA^	4.10 ± 0.52^bdA^	3.85 ± 0.42^abA^
	60 kV	4.32 ± 0.16^bA^	4.20 ± 0.21^bAB^	3.72 ± 0.36^cAB^	3.67 ± 0.56^bAB^	3.48 ± 0.58^bB^
	70 kV	3.63 ± 0.28^cA^	3.54 ± 0.42^cA^	3.31 ± 0.37^cAB^	2.72 ± 0.50^cB^	2.55 ± 0.47^cB^

Treatments involve a control consisting of washed pork muscle without myoglobin (control) and a mixture of washed pork muscle plus myoglobin and after that processed at different voltages (0, 50, 60, 70 kV for 180 s).

A-B means without common superscripts in a row are significantly different (*p* < 0.05).

a-d means without common subscripts in a column within a parameter are significantly different (*p* < 0.05). Replicates per treatment were *n* = 3.

#### 3.1.2. Non-heme iron contents

**Figure 1:**
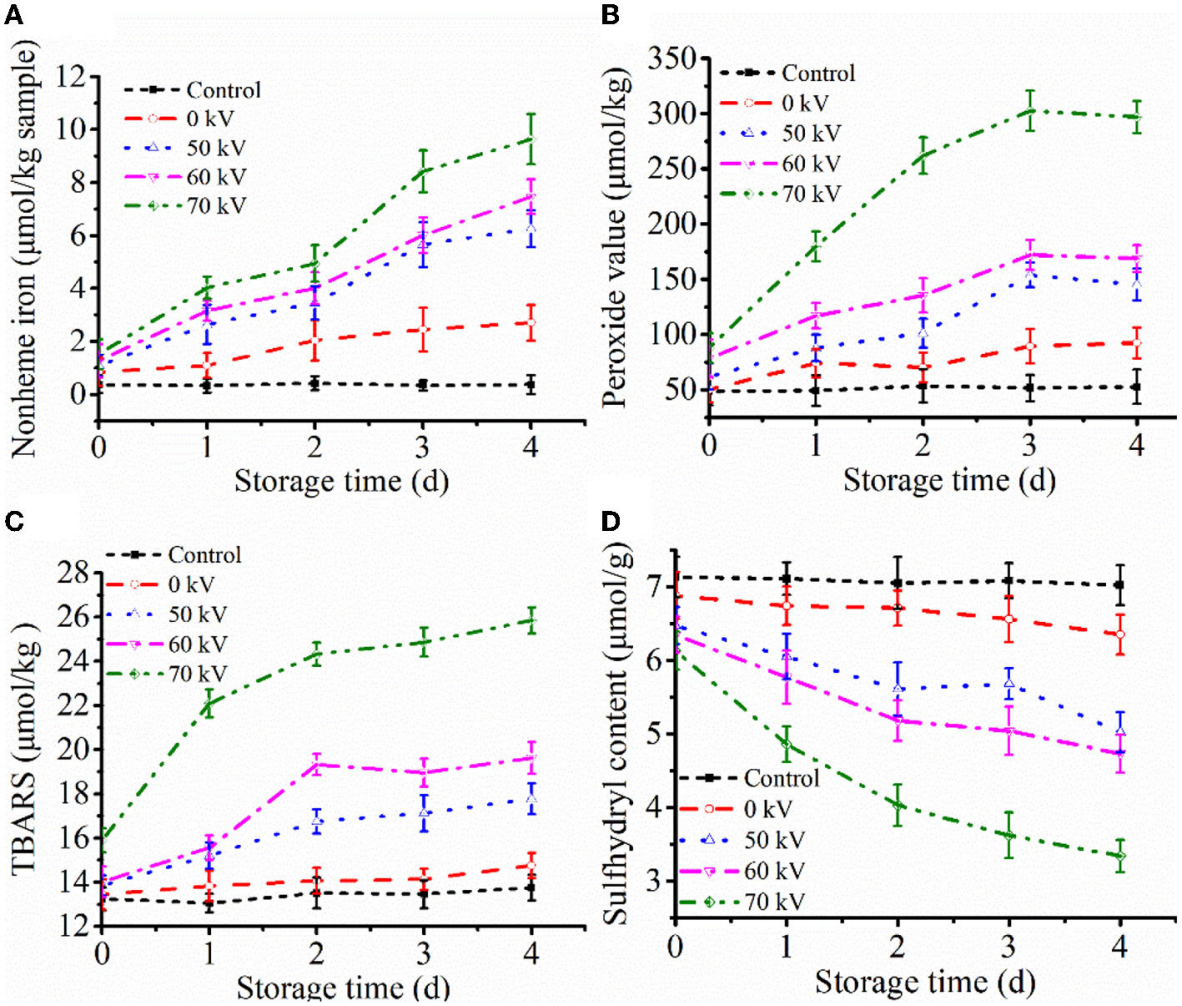
Profiles of **(A)** non-heme iron contents, **(B)** peroxide value (PV), **(C)** thiobarbituric acid reactive substances (TBARS), and **(D)** total sulfhydryl group content (T-SH) of washed pork muscle added without and with added Mb at pH 7.0 after treatment by DBD-CP at 4°C for 4 days. Control, washed pork muscle; 0 kV, washed pork muscle added Mb; The remaining groups were treated by DBD-CP at 50, 60, 70 kV for 180 s, respectively; The operating frequency was 50 Hz. Replicates per treatment were *n* = 3. Means and standard deviations are shown.

Heme degradation can be represented by increasing non-heme iron content (23). Non-heme contents in washed muscle during storage with DBD-CP treatment are shown in Figure 1A. No notable change in content was obtained in the control, and the mean value ranged from 0.30–0.50 μmol/kg muscle during storage (*p* ≥ 0.05). Obviously, non-heme content in the treated groups increased significantly (*p* < 0.05) (0 kV: 0.84 to 2.71, 50 kV: 1.08 to 6.26, 60 kV: 1.27 to 7.47, 70 kV: 1.52 to 9.64 μmol/kg muscle) in 4 days. The results suggested that DBD-CP could markedly promote the release of non-heme iron. Furthermore, the denaturation of the porphyrin ring of Mb possibly occurred under cold plasma with high voltage conditions, causing the release of free iron called “non-heme iron.” In WPM-Mb, iron ions continued to accumulate during time. It has also been demonstrated that H_2_O_2_/O${}_{2}^{-}$ radicals generated by DBD-CP are responsible for heme degradation (24). The results correlated with previous research (25, 26), which found that H_2_O_2_ could facilitate heme degradation and release free iron in the gastric fluid model. Another study illustrated that H_2_O_2_, OH radicals, and UV radiation produced by CP treatment accelerated the heme degradation of horseradish peroxidase protein (27). Hence, DBD-CP treatment might be the main factor accelerating the release of free iron ions and the heme degradation from myoglobin.

#### 3.1.3. PV

PV is an essential parameter that evaluates the extent of the primary stage of lipid oxidation (28). As seen in Figure 1B, for the control, no significant changes were found in PV during the entire storage at 4°C (*p* ≥ 0.05). Washing processes may remove water-soluble antioxidants, pro-oxidants, and some lipids from the muscle, thereby eliminating the interfering reactions caused by these factors (29). Consequently, lipid oxidation was negligible in washed muscle. In the WPM-Mb, the formation of lipid peroxide in the sample (0–60 kV) was slow between day 0 to day 3 (*p* < 0.05), while the 70 kV sample increased rapidly in the same period (*p* < 0.05), and followed by a stable trend by day 4 (*p* ≥ 0.05) (Figure 1B). In addition, with the increase of the voltage, the PV of the WPM-Mb was prominently increased (*p* < 0.05). The data showed that DBD-CP significantly enhanced PV in WPM-Mb (50-70 kV), indicating that DBD-CP promoted Mb-induced lipid oxidation in the washed muscle. MetMb could form ferryl-Mb to facilitate lipid oxidation (30).

#### 3.1.4. TBARS

TBARS during storage is depicted in Figure 1C. The TBARS of control were not significantly changed (*p* ≥ 0.05). For WPM-Mb (0 kV), a gradual increase (*p* ≥ 0.05) in TBARS was found during 0–3 days, followed by a notable increase after 3 days (*p* < 0.05). During the first 2 days, TBARS values in WPM-Mb (50–70 kV) were found to increase rapidly (*p* < 0.05), and no notable change was observed after that (*p* ≥ 0.05). During storage, WPM-Mb (50–70 kV) generally had higher TBARS values, compared with 0 kV samples (*p* < 0.05). Although the TBARS of all samples treated by DBD-CP increased with storage time, the rate of increase was higher for 70 kV samples than for 50–60 kV samples. Therefore, lipid oxidation catalyzed by Mb could be promoted *via* DBD-CP treatment, especially a higher voltage. What's more, it should be noted that PV and TBARS increased with increased voltage even without any storage of the washed muscle matrix containing Mb (e.g., 0-time data). This result suggested that free radical species and oxidants generated during 180 s of exposure caused considerable lipid oxidation. A possible good explanation was that radicals produced during DBD-CP processing could facilitate the generation of peroxides and lipid oxidation, increasing TBARS (31). The data was coincident with the changes of a^*^-value and PV. Additionally, it was inferred that the myoglobin oxidation and the conformational of Mb might be induced by radicals produced by DBD-CP, leading to the promotion of lipid oxidation in WPM (31).

#### 3.1.5. Total free sulfhydryl content

Changes of T-SH contents in WPM are represented in Figure 1D. It can be seen that the T-SH contents of control and 0 kV samples were about 7.2 and 6.7 μmol/g muscle, respectively, and had no remarkable differences (*p* ≥ 0.05) at 0 to 4 days. The content of T-SH in WPM-Mb gradually decreased with the increase of voltages (50–70 kV) over the storage time (*p* < 0.05). The results demonstrated that DBD-CP could promote T-SH oxidation, and the higher the voltage intensity, the more the number of T-SH was oxidized. What's more, Feng et al. (32) pointed out that sulfhydryl groups on the surface of proteins could be readily converted into disulfide bonds when attacked by highly oxidizing active substances (e.g., H_2_O_2_ and ROS). Thus, the loss of thiols in the washed pork muscle could be due to cysteine oxidation in the myofibrillar proteins and/or myofibrillar protein disulfide formation.

### 3.2. The structural properties of myoglobin

#### 3.2.1. Protein patterns

**Figure 2:**
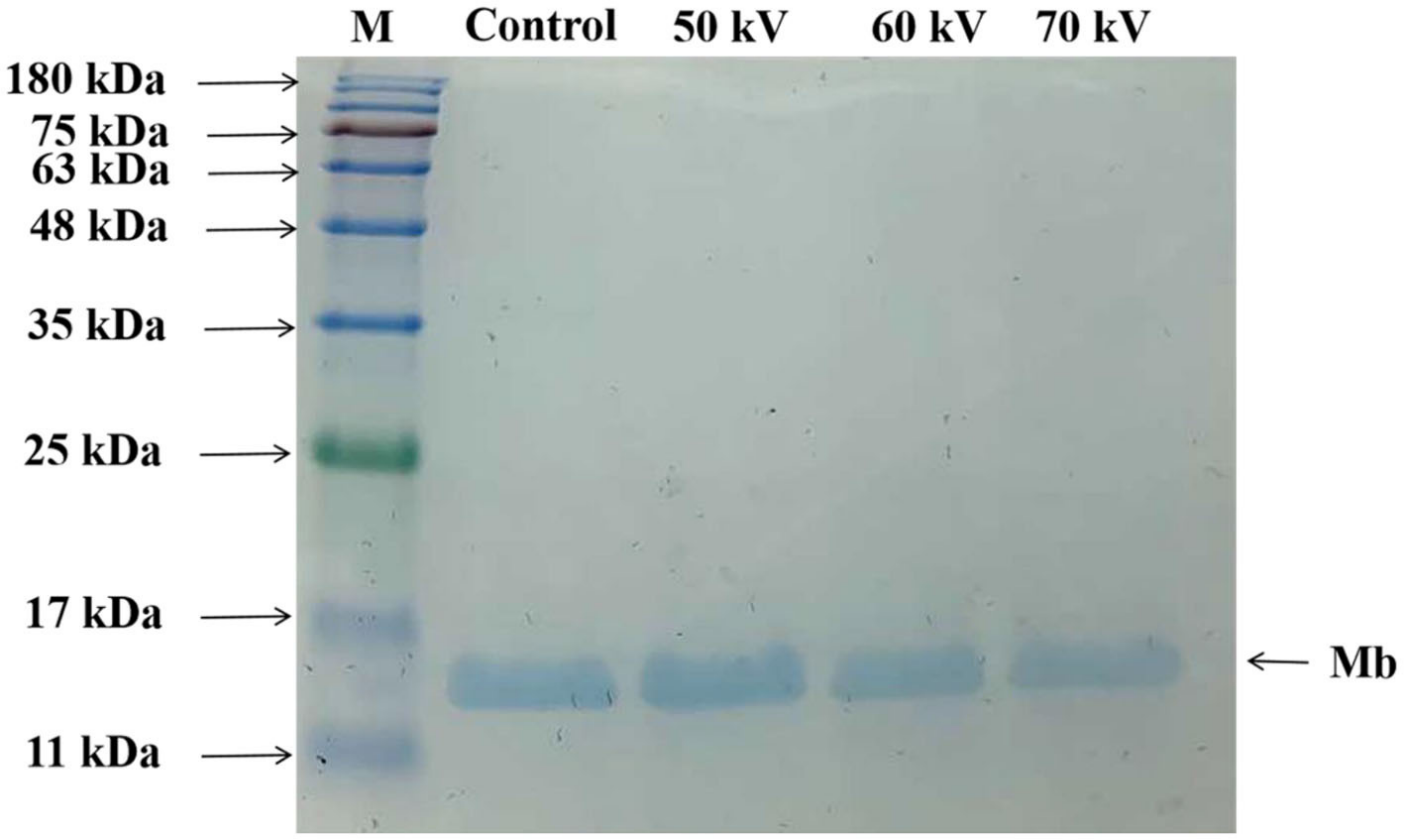
Sodium dodecyl sulfate-polyacrylamide gel electrophoresis (SDS-PAGE) patterns of myoglobin treated by DBD-CP at 50, 60, and 70 kV. Control, without treatment; M, molecular standards.

SDS-PAGE was employed for characterizing the protein patterns of Mb. As depicted in Figure 2, most of the bands were concentrated around 17 kDa. The results showed that no noticeable loss or increase of Mb bands accompanied different voltage treatments, indicating that the molecular weight of Mb almost did not change, and no crosslinked Mb was produced after DBD-CP treatment. The result was consistent with previous research, which suggested no significant change in the molecular weight or MP bands after atmospheric pressure plasma jet processing (33). Ji et al. (34) also outlined that the application of CP at different times (1-4 min) did not alter the electrophoretic profile of peanut protein. In our study, DBD-CP caused no distinct changes in the SDS-PAGE shape between untreated and treated groups.

#### 3.2.2. The absorption spectra and autoxidation of Mb

**Figure 3:**
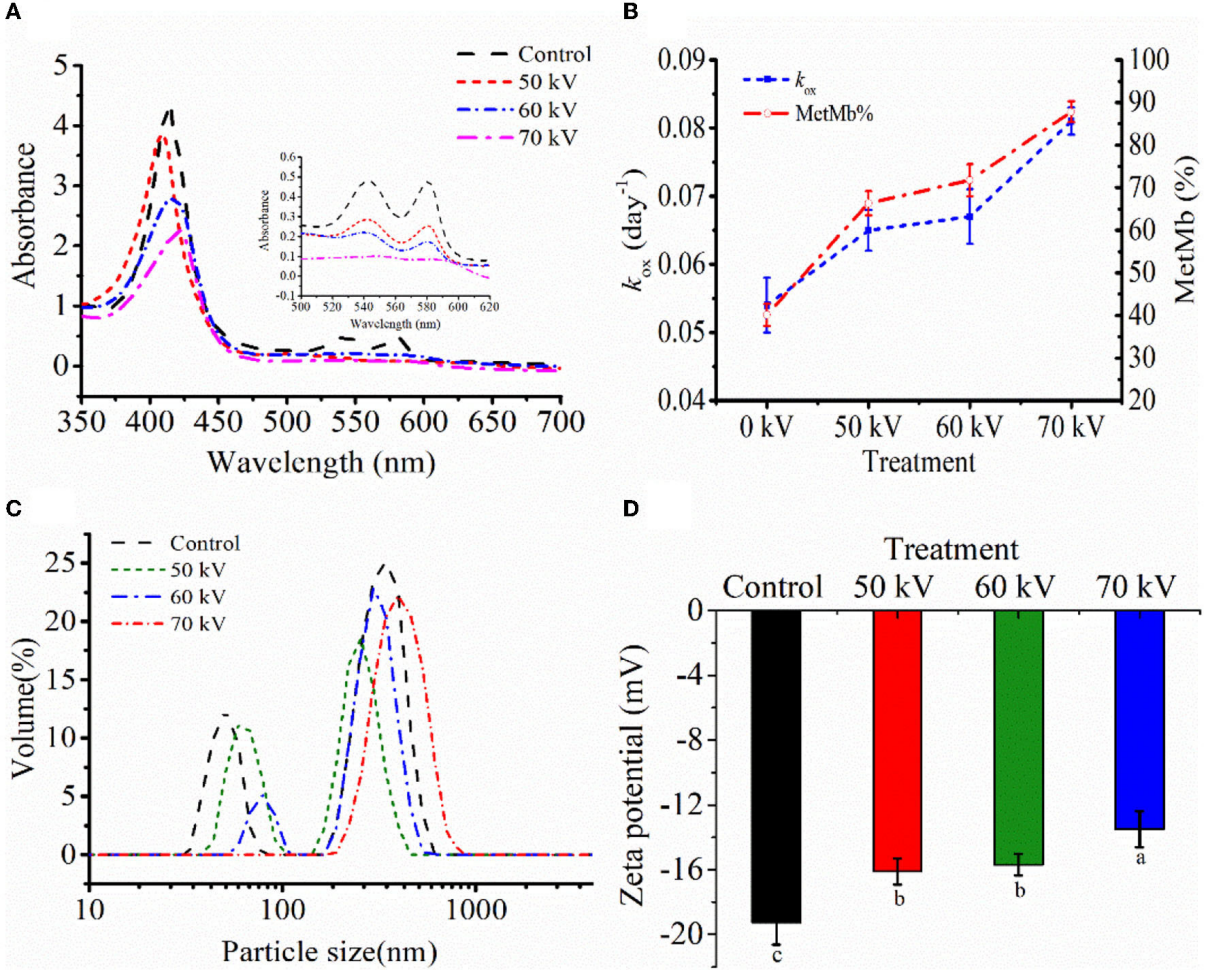
**(A)** The UV absorption. **(B)** metMb proportion and autoxidation rate (*k*_*ox*_), **(C)** particle size distribution, and **(D)** zeta potential of myoglobin with and without DBD-CP treatment. *k*_*ox*_is equivalent to the probability of decay of a single molecule to the ferric form in 4 days. Note: Control, protein without DBD-CP treatment; The remaining groups were treated by DBD-CP at 50, 60, 70 kV, and 50 Hz for 180 s, respectively. The operating frequency was 50 Hz. Replicates per treatment were *n* = 3. Means and standard deviations are shown.

Figure 3A shows the absorbance spectra of Mb untreated and treated with DBD-CP. Fully reduced oxyMb usually has the Soret peak at 415–418 nm. In the prepared Mb solution, the intense peaks in the blue region (350–450 nm) corresponding to the Soret bands were found at 412 nm for oxyMb, which gradually decreased with DBD-CP treatment voltages, indicating that the content of oxyMb reduced and/or the structure might change (Figure 3A). Also, for the control treatment, a deeper valley was found between 600 and 550 nm, it might be attributed to the formation of oxyMb (Figure 3A). The valley decreased with the increase of the treatment voltage and disappeared at 70 kV. The spectra were more characteristic of small amounts of metMb, some degraded heme (as suggested by the non-heme iron determinations), and some intact heme that had been released from the globin forming some apoMb (the Soret peak absorbance decreased from about 4.21 to 2.20). The non-heme iron results just after DBD-CP indicated about 1.5 μmol/kg for the 70 kV treatment (Figure 1A), equivalent to about 4% degraded heme and 96% remaining intact heme. So, this data suggested DBD-CP released considerable amounts of heme from the globin that was still intact. And the heme to promote lipid oxidation has been described (35).

As depicted in Fig. 3B, results were expressed as autoxidation rates of Mb during 4 days and the relative metMb proportion at day 4. Mb with DBD-CP treatment autoxidized faster than untreated samples. DBD-CP treatment significantly accelerated metMb formation, especially 70 kV samples (*p* < 0.05), which also coincided with the changes in absorption spectra (Figure 3A). This result might be attributed to that electrons generated after DBD-CP treatment combining with the ligand-O_2_ in the ferrous iron of oxyMb, leading to the formation of metMb (4). After treatment, the metMb proportion remarkably increased (*p* < 0.05) with the treatment voltages at day 4. The increase of metMb ratio together with *k*_*ox*_(Figure 3B) demonstrated that ferrous myoglobin species (oxyMb, deoxyMb) was oxidized to form ferric metMb during storage (36). In brief, DBD-CP facilitated the oxidation of ferrous myoglobin, and the oxidation rate increased with the increase of voltage intensity.

#### 3.2.3. Particle size and zeta potential distribution

To further characterize the overall distribution and aggregation of the protein after DBD-CP treatment, particle size and zeta potential were tested. In Figure 3C, the Mb treated by DBD-CP exhibited a better size distribution than the control, which showed an apparent right shift of the size distribution peak with the increasing voltages (Figure 3C). The initial Mb was characterized as a high contribution of a larger size (aggregates) zone (~1000 nm) and followed by a smaller size zone (~100 nm). After treatment at 50 and 60 kV, the particle size distribution of the smaller zone was notably reduced. Upon 70 kV treatment, the smaller zone disappeared, and the aggregate particle was extensively promoted. The data could be ascribed to the fact that (i) DBD-CP might accelerate intermolecular aggregation and alter the size distribution of myoglobin; (ii) Mb solution was bombarded by high-energy particles (e.g., electrons, ions, and other active species), resulting in the exposure of functional groups of Mb and increased its structural instability (34).

As shown in Figure 3D, when the voltage increased from 0 to 70 kV, the potential changed from 19.26 ± 1.38 to 13.50 ± 1.11 mV. These changes in the surface charge of Mb could reduce the intermolecular repulsion force and facilitate the aggregation of the protein. The variation trend of zeta potential was coincident with the variation trend of particle size distribution. Therefore, it could be inferred that partial aggregation occurred between Mb molecules under the DBD-CP treatment.

#### 3.2.4. CD spectroscopy

**Figure 4:**
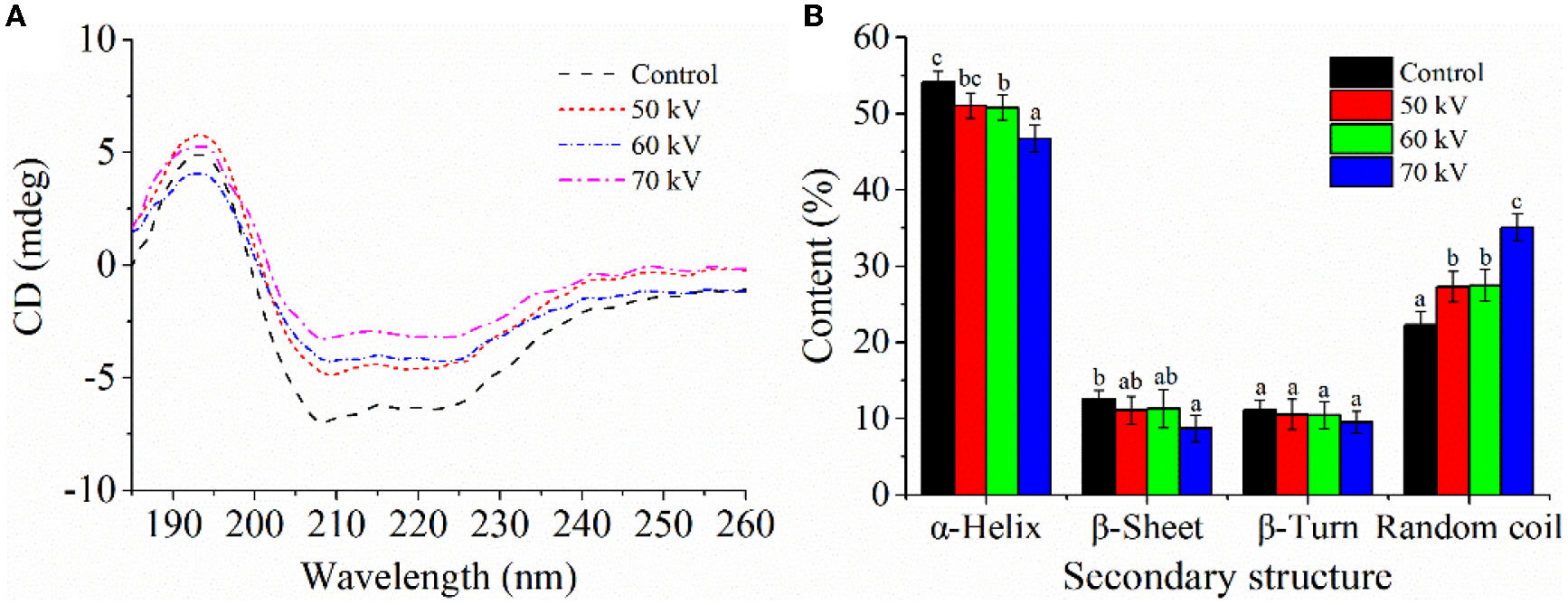
Effect of DBD-CP on the secondary structure of myoglobin. **(A)** Circular dichroism (CD) spectra of Mb treated by different voltage. **(B)** The secondary structural changes of myoglobin were calculated from CD spectra; each sample was analyzed in triplicate; different small letters indicate significant differences (*p* < 0.05).

The CD spectra of Mb after DBD-CP processing is shown in Figure 4A, and the secondary structures calculated according to the spectra are shown in Figure 4B. In Figure 4A, the characteristic negative bands of the α-helix appeared at around 208 and 222 nm, implying that α-helix was the major secondary structure of myoglobin, which was consistent with previous research (37). The negative peaks at 208 and 222 nm were shifted upward by the DBD-CP treatment, which reflected losses of α-helix structure in Mb. In Figure 4B, DBD-CP caused a remarkable decrease in the proportion of α-helix (54.1%, control to 46.75%, 70 kV), but a noticeable increase in random coil proportion (22.3%, control to 35.05%, 70 kV) with increasing treatment voltages (*p* < 0.05). The results suggested that the secondary structure of Mb was damaged by DBD-CP, which made the structure disordered. The disordered structure was enhanced with the treatment of 50 to 70 kV, in which the notable changes occurred in α-helix and random coil. According to reports, proteins could maintain their bioactivity through weak interactions such as hydrophobic, hydrogen, or ionic bonds (38). In the present study, DBD-CP processing could break some Mb hydrogen bonds and affect the α-helix structure, weakening the tensile strength. Thus, the interaction of Mb was altered by DBD-CP treatment, which facilitated the partial aggregation of Mb. This result might reveal that the aggregation between Mb proteins could be mediated by protein oxidation, which coincided with metMb content and particle size changes (15).

## 4. Conclusion

In summary, DBD-CP treatment, especially at 70 kV, caused a significant decrease in the redness and the total sulfhydryl content, while increased non-heme content and facilitated Mb-induced lipid oxidation in washed pork muscle throughout storage. The results can be explained from two aspects: (i) the conformation of Mb is altered by DBD-CP treatment; (ii) reactive species in DBD-CP promote Mb oxidation and lipid oxidation. Meanwhile, the changes of absorption spectra and autoxidation rate of Mb further confirm that reactive species produced by DBD-CP treatment promotes some release of heme from the globin, forming some apoMb. This process leads to lipid oxidation and the aggregation of some metMb and apoMb molecules, which affects the particle size distribution and the zeta potential. More importantly, with the increase of treatment voltages (0–70 kV), the structural change of Mb becomes more apparent. To sum up, DBD-CP accelerates the lipid oxidation in WPM-Mb mainly due to the reaction of reactive species in DBD-CP to promote the Mb oxidation and change the Mb conformation. Therefore, inhibition of lipid oxidation in pork or other muscle food systems containing heme proteins needs further studies to be performed in the future.

## Data availability statement

The raw data supporting the conclusions of this article will be made available by the authors, without undue reservation.

## Author contributions

XW and JW: data curation, writing-original draft, and data collection and analysis. ZW: data curation. WY, HZ, JZ, and JW: supervision and writing-review and editing. All authors have read and agreed to the published version of the manuscript.
